# Public acceptance of COVID-19 control measures and associated factors during Omicron-dominant period in China: a cross-sectional survey

**DOI:** 10.1186/s12889-024-17646-3

**Published:** 2024-02-21

**Authors:** Jiayi Zhong, Qianhong Zhong, Husheng Xiong, Dawei Wu, Caiyun Zheng, Shuang Liu, Qinyi Zhong, Yan Chen, Dingmei Zhang

**Affiliations:** 1 School of Public Health, Sun Yat-Sen University, 510080 Guangzhou, Guangdong China; 2 Department of Tuberculosis Control, The Fourth People’s Hospital of Foshan city, 528000 Foshan, Guangdong China; 3 School of Law, Sun Yat-Sen University, 510080 Guangzhou, Guangdong China; 4 Medical College of Shaoguan University, 512026 Shaoguan, Guangdong China

**Keywords:** Public acceptance, COVID-19, Control measures, Omicron variant, Vaccination, Public health strategy

## Abstract

**Objectives:**

This study aims to evaluate the public acceptance of coronavirus disease 2019 (COVID-19) control measures during the Omicron-dominant period and its associated factors.

**Methods:**

A cross-sectional design was conducted and 1391 study participants were openly recruited to participate in the questionnaire survey. Logistic regression model was performed to assess the association between the public acceptance and potential factors more specifically.

**Results:**

By August 26, 2022, 58.9% of the study participants were less acceptive of the control measures while 41.1% expressed higher acceptance. Factors associated with lower acceptance included young age, such as < 18 (OR = 8.251, 95% CI: 2.009 to 33.889) and 18–29 (OR = 2.349, 95% CI: 1.564 to 3.529), and household per capita monthly income lower than 5000 yuan (OR = 1.512, 95% CI: 1.085 to 2.105). Furthermore, individuals who perceived that the case fatality rate (CFR) of severe acute respiratory syndrome coronavirus-2 (SARS-CoV-2) was very low (OR = 6.010, 95% CI: 2.475 to 14.595) and that the restrictions could be eased once the CFR dropped to 2–3 times of the influenza (OR = 2.792, 95% CI: 1.939 to 4.023) showed greater oppositional attitudes. Likewise, respondents who were dissatisfied with control measures (OR = 9.639, 95% CI: 4.425 to 20.998) or preferred fully relaxation as soon as possible (OR = 13.571, 95% CI: 7.751 to 23.758) had even lower acceptability. By contrast, rural residents (OR = 0.683, 95% CI: 0.473 to 0.987), students (OR = 0.510, 95% CI: 0.276 to 0.941), public (OR = 0.417, 95% CI: 0.240 to 0.727) and private (OR = 0.562, 95% CI: 0.320 to 0.986) employees, and vaccinated participants (OR = 0.393, 95% CI: 0.204 to 0.756) were more compliant with control measures.

**Conclusion:**

More than half of the Chinese public were less supportive of COVID-19 control measures during Omicron-dominant period, which varied based on their different demographic characteristics, cognition and overall attitude towards SARS-CoV-2 infection. Control measures that struck a balance between public safety and individual freedom would be more acceptable during the pandemic.

**Supplementary Information:**

The online version contains supplementary material available at 10.1186/s12889-024-17646-3.

## Introduction

Since the emergence of Omicron variant in November 2021, due to its higher risk of reinfection, faster doubling time and stronger immune escape ability [1–3], COVID-19 pandemic in 2022 has further swept across China. At that time, the Chinese government adhered to a “dynamic COVID-zero strategy” to curb this highly contagious variant [4]. Nevertheless, in the face of Omicron epidemic in late February 2022 in Shanghai, China, precise control by grid management in dynamic COVID-zero strategy was not so effective and began to expose limitations [5, 6].

The formulation and implementation of interventions related to behavior change were highly dependent on the cooperation and compliance of all members of society, which was well reflected during the severe acute respiratory syndrome (SARS) epidemic in China [7–9]. The Chinese people at that time were more compliant and willing to accept the control measures, which were necessary for China to defeat SARS finally. In contrast, countries around the world had also adopted some control measures during the COVID-19 pandemic, such as wearing of face masks, practicing hand hygiene, and leveraging big data and digital technology for contacts tracing [10]. Moreover, respondents from Malaysia, Pakistan and Singapore also showed a high cooperation with these measures [11–13].

However, as COVID-19 pandemic continued to evolve, more citizens began to attach importance to privacy and personal freedoms. In March 2022, Shanghai still stuck to the dynamic COVID-zero strategy amid the large-scale Omicron epidemic [14], but the indefinite extension of the harsh lockdowns also triggered anxiety and panic among Shanghai residents [15]. Then, broader dissension has emerged among Chinese public for the “dynamic COVID-zero” policy [16], which has begun to show irresistible resistance in mainland China. Hence, in the face of the huge challenge posed by Omicron variant, the control measures must be taken more prudently, especially fully considering the public’s acceptance of relevant policies.

To date, although restrictions have been eased since December 2022 in China [17], there was still a lack of surveys concerning Chinese citizens’ general attitudes toward control measures during the dynamic COVID-zero period. Consequently, this study investigated the public acceptance of COVID-19 control measures and associated factors in China in August 2022. These past experiences played a significant role in deciding present mental and behavioral state of the respondents and conferring an empirical basis for the formulation of future public health strategies.

## Methods

### Study design and participants

This study adopted a cross-sectional design to publish a self-designed questionnaire on sojump (https://www.wjx.cn/, the most commonly used online survey tool in China) and aimed to investigate Chinese citizen’s acceptance of COVID-19 control measures and related factors during the Omicron-dominant period. We conducted an open online survey to collect the data via convenience sampling since it was not feasible to do a community-based national sampling survey during the dynamic COVID-zero period.

Participants in this study were recruited primarily through WeChat, the largest social media program in China. Specifically, the recruitment started with clicking the link or scanning the quick response code (QR code) posted on our WeChat Moments (called Peng-You-Quan in China) and shared in group chats, where the respondents could browse and come across the web-based questionnaire. After finishing, participants could also voluntarily share and repost the questionnaire to their WeChat Moments or friends, which further expanded the scope of recruitment. To guarantee the reliability and generalizability of the results, all participants were anonymous and did not receive any honorarium during the whole recruitment process.

The data was collected from August 14, 2022 to August 26, 2022 and a total of 1402 questionnaires were collected finally. After careful inspection, 11 unreasonable invalid questionnaires (missing information) were eliminated. This study finally analyzed a total of 1391 valid questionnaires, of which the recovery rate was 99.2%.

### Survey questionnaires

The anonymous questionnaire consisted of four parts. The first part collected demographic information, such as sex, age, residency, educational qualification, underlying diseases, occupation, household per capita monthly income, history of a lockdown or controlled area and history of SARS-CoV-2 infection. In accordance with the COVID-19 Prevention and Control Plan (Ninth edition) released on June 28, 2022, which was also applicable until August 2022, the lockdown and controlled areas referred to residential areas with SARS-CoV-2 positive cases and areas where there existed the risks of transmission. All residents in the lockdown area were prohibited from leaving their homes, so they were provided with quantitative real-time polymerase chain reaction (qPCR) test for SARS-CoV-2 and household supplies by specialized workers. In contrast, all residents in the controlled area were strictly forbidden to leave the community in principle. Nevertheless, daily SARS-CoV-2 testing and the purchase of household goods could be obtained by unified arrangement, without the need for specialized staff to visit their homes.

The second part investigated the public’s cognition and overall attitude towards SARS-CoV-2 infection and control measures, including the fear of SARS-CoV-2 infection (0−10 points, where 0 was no fear at all and 10 was very scared), perceived severity of SARS-CoV-2, COVID-19 vaccination status, overall satisfaction with control measures and future expectations. The third part surveyed the impact of control measures on public’s life (11 items) and work (5 items). In this part, participants could choose two options: “Yes” or “No”. When a participant selected “no”, it was scored as zero, and when the answer was “yes”, it was scored as one point. Ultimately, the total score of the impact on public’s life and work was calculated separately. Based on the lower quartile as a cut-off to distinguish different total score groups [18, 19], higher total score was considered as the greater impact of control measures on the public’s life or work.

**Fig. 1:**
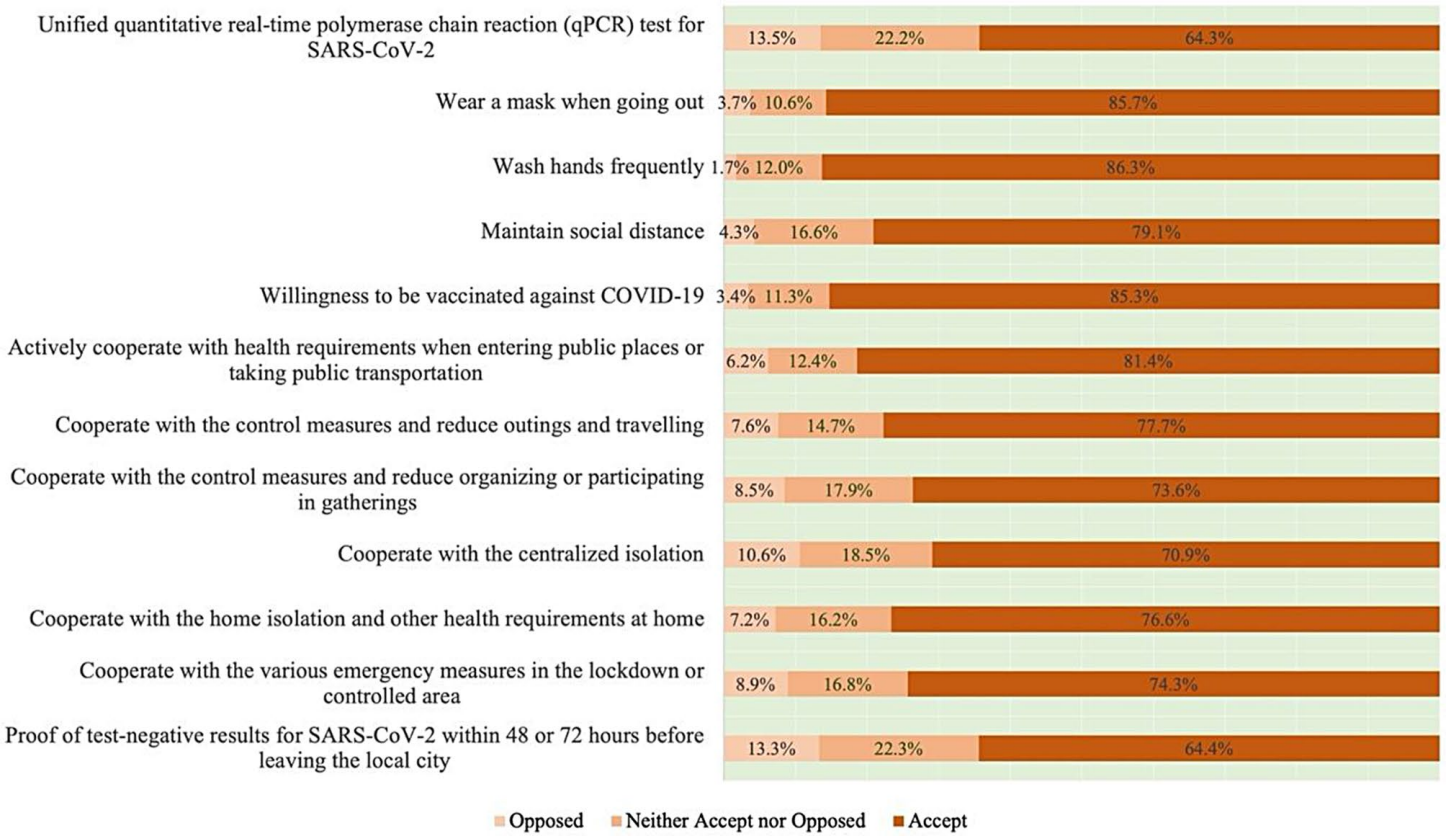
Public acceptance of 12 specific control measures during the Omicron-dominant period in China, August 2022

The fourth part was a survey on the public acceptance of COVID-19 control measures. In this section, participants selected their acceptance of 12 specific measures implemented during the Omicron-dominant period. The acceptance proportions that the public selected for each measure were presented in Fig. 1. For each specific measure, acceptance levels were divided into “Accept”, “Neither Accept nor Opposed” and “Opposed” from high to low, of which the score was corresponded to zero, one point and two points respectively. For instance, when a respondent chose the “Accept” answer, he / she would receive zero. Otherwise, it would be scored as one point or two points with the options corresponded to “Neither Accept nor Opposed” or “Opposed”, respectively. Finally, the sum of the points of 12 measures was recorded as the total score of acceptance for each participant, with the minimum of 0 and the maximum of 24 points.

As the calculated rules mentioned above, participants with higher total scores had lower acceptance of COVID-19 control measures. It could be further explained that a total score of zero (lower quartile as a cut-off) was defined as high public acceptance of COVID-19 control measures (i.e., the participants selected “Accept” for all 12 measures), while a total score of one point and above was defined as low acceptance (i.e., the participants selected "Neither Accept nor Opposed" or “Opposed” for at least one of the 12 measures).

Before formal study, a pilot study was conducted for the web-based questionnaire to ensure that the questions we set were understandable and applicable to the public in various regions of China. The questionnaire was released after being reviewed by professors and professionals to ensure facial validity. Reliability analysis showed that the Cronbach's alpha of the impact on public’s life was 0.861, and that of the public’s acceptance was 0.948, indicating that the questionnaire had good reliability.

### Statistical analysis

The data of this survey derived from Sojump. Continuous variables were described by the mean and standard deviation (SD), or median and interquartile range (IQR) respectively. Categorical variables were described by percentage (%).

In addition, we also conducted univariate regression analysis to initially evaluate potential factors that might affect the public’s acceptance of control measures, including demographic characteristics, public’s cognition and overall attitude, and the impact of control measures on public’s life. Notably, since some respondents like students were not yet formally engaged in work, they were unable to answer questions about the impact on work. Hence, the total score of this part was not included in the final multiple logistic regression analysis.

Finally, the variables with *P* < 0.1 in the univariate regression analysis were included in the multiple logistic regression analysis [20], where *P* < 0.05 was regarded as a significant difference. Associations were estimated and interpreted with odds ratio (OR) and 95% confidence interval (95% CI). All data was processed by SPSS software (version 26.0) for statistical analysis.

## Results

### Sample characteristics

A total of 1391 valid questionnaires were analyzed in this study. 58.9% of the participants had a low acceptance of control measures (a total score of one point and above, 819 of 1391), while 41.1% were highly acceptive (a total score of zero, 572 of 1391). Of the 1391 respondents, 560 (40.3%) were male and 831 (59.7%) were female. A total of 50.7% of participants were aged between 30 and 49 years, and the participants with low acceptance were younger than that with high acceptance (32.61 years vs. 35.56 years, *P* = 0.036).

**Fig. 2:**
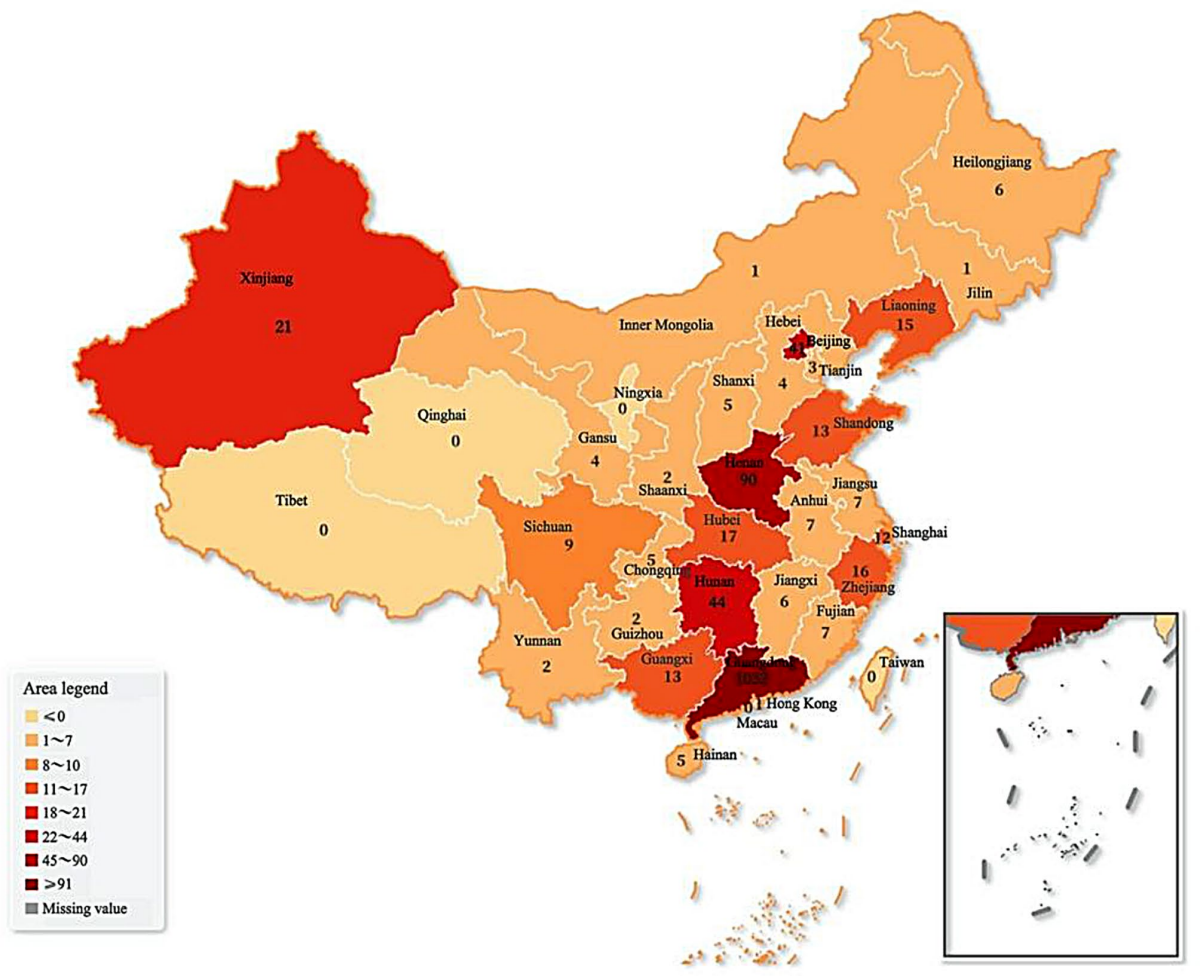
Geographical distribution of the study participants

Furthermore, the majority of participants lived in urban areas (86.1%), had no underlying diseases (91.7%), had graduated from universities (49.7%) and were working in the public sector (43.9%) or private sector (24.3%), with some students (19.3%) by occupation. With regard to household income, 66.6% of the respondents reported that the per capita monthly in their families was lower than 12,000 yuan. Characteristics and geographical distribution of the included participants were shown in Table 1; Fig. 2.

**Table 1:** Demographic characteristics of the study participants, August 2022

Possible Variables	Total Participants (*n* = 1391) (%)	Public Acceptance			Univariate logistic regression analysis	
		High (*n* = 572) (%)	Low (*n* = 819) (%)	*P*-Value ^1^	Odds Ratio (OR) and 95% Confidence Interval (95% CI)	*P*-Value ^2^
Sex				0.485		0.485
Male	560 (40.3)	224 (39.2)	336 (41.0)		1.081 (0.869, 1.344)	
Female	831 (59.7)	348 (60.8)	483 (59.0)		Reference	
Age (years), median (interquartile range)	34.01 (25.06, 42.28)	35.56 (25.70, 42.84)	32.61 (24.91, 42.05)	0.036 *	-	-
Age category (years)				0.039 ^a^*		0.042 *
< 18	8 (0.6)	2 (0.4)	6 (0.7)		2.328 (0.451, 12.013)	
18–29	559 (40.2)	206 (36.0)	353 (43.1)		1.330 (0.891, 1.986)	
30–49	705 (50.7)	312 (54.5)	393 (48.0)		0.978 (0.661, 1.446)	
≥ 50	119 (8.5)	52 (9.1)	67 (8.2)		Reference	
Residency				< 0.001 ***		< 0.001 ***
Rural	193 (13.9)	106 (18.5)	87 (10.6)		0.523 (0.389, 0.702)	
Urban	1198 (86.1)	466 (81.5)	732 (89.4)		Reference	
Educational qualification				< 0.001 ***		< 0.001 ***
High school or below	207 (14.9)	121 (21.2)	86 (10.5)		0.337 (0.261, 0.436)	
Universities and colleges	692 (49.7)	298 (52.1)	394 (48.1)		0.622 (0.522, 0.741)	
Master degree or above	492 (35.4)	153 (26.7)	339 (41.4)		Reference	
Underlying diseases				0.954		0.731
No	1276 (91.7)	525 (91.8)	751 (91.7)		Reference	
Yes	115 (8.3)	47 (8.2)	68 (8.3)		0.952 (0.719, 1.260)	
Occupation				0.005 **		0.005 **
Public sector	610 (43.9)	237 (41.4)	373 (45.5)		1.180 (0.655, 2.127)	
Private sector	338 (24.3)	146 (25.6)	192 (23.5)		0.986 (0.538, 1.807)	
Self-employed or businessman	126 (9.0)	70 (12.2)	56 (6.8)		0.600 (0.308, 1.168)	
Students	268 (19.3)	98 (17.1)	170 (20.8)		1.301 (0.701, 2.414)	
Unemployed or retired	49 (3.5)	21 (3.7)	28 (3.4)		Reference	
Household per capita monthly income (yuan)				0.003 **		< 0.001 ***
< 5000	388 (27.9)	177 (30.9)	211 (25.8)		0.703 (0.548, 0.902)	
5000–11,999	539 (38.7)	236 (41.3)	303 (37.0)		0.715 (0.567, 0.901)	
12,000–20,000	242 (17.4)	81 (14.2)	161 (19.7)		1.102 (0.838, 1.448)	
> 20,000	222 (16.0)	78 (13.6)	144 (17.6)		Reference	
History of a lockdown area				0.063		0.004 **
No	1236 (88.9)	519 (90.7)	717 (87.5)		Reference	
Yes	155 (11.1)	53 (9.3)	102 (12.5)		1.461 (1.127, 1.894)	
History of a controlled area				0.155		0.025 *
No	1050 (75.5)	443 (77.4)	607 (74.1)		Reference	
Yes	341 (24.5)	129 (22.6)	212 (25.9)		1.236 (1.028, 1.487)	
History of SARS-CoV-2 infection				0.148 ^a^		0.999
No	1387 (99.7)	572 (100.0)	815 (99.5)		Reference	
Yes	4 (0.3)	0 (0.0)	4 (0.5)		0.000	

*Note*: *P-*values comparing different groups were from t-test, chi-squared test and univariate logistic regression analysis. ^a^ Fisher’s exact test. ^1^ Significance difference: *P* < 0.05. ^2^ Significance difference of univariate logistic regression analysis: *P* < 0.1. * *P* < 0.05. ** *P* < 0.01. *** *P* < 0.001. The “-” indicated no data

### Cognition and overall attitude towards SARS-CoV-2 infection and control measures

Generally, the respondents showed a moderate fear about SARS-CoV-2 infection (4.78±3.19 scores), and low-acceptance individuals were less afraid of contracting SARS-CoV-2 than the high-acceptance (4.43 scores vs. 5.29 scores, *P* < 0.001). By the end of August 2022, 59.5% of respondents perceived that the case fatality rate (CFR) of SARS-CoV-2 kept a low or extremely low level. Moreover, 88.0% of them reputed that the restrictions could be appropriately eased when the CFR reduced to one-tenth or equal to influenza. Meanwhile, the vast majority of respondents (97.2%, 1352 of 1391) had been vaccinated against COVID-19 before (Table 2).

**Fig. 3:**
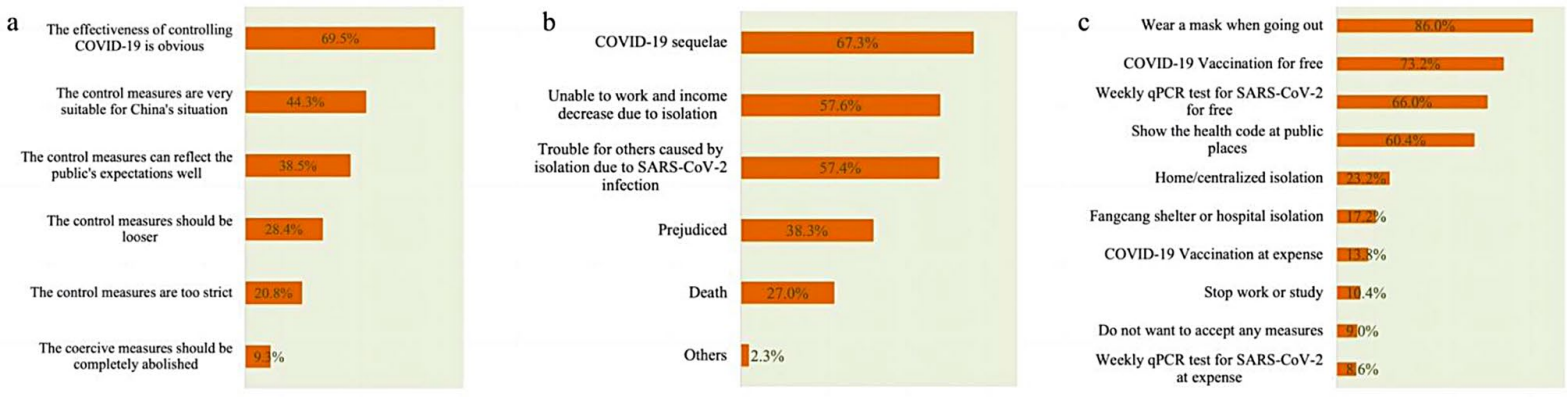
Multiple-answer questions regarding public’s cognition and overall attitude towards SARS-CoV-2 infection and control measures in China, August 2022. (**a**) Public’s evaluations of COVID-19 control measures. (**b**) Public’s biggest concern if infected with SARS-CoV-2. (**c**) Control measures acceptable to the public if the case fatality rate of SARS-CoV-2 was not higher than that of influenza

In this survey, over 75% of the participants were satisfied or very satisfied with the control measures taken previously. Most respondents deemed that the control measures curbed the epidemic effectively (69.5%), conformed to China’s actual situations (44.3%) and reflected the public’s expectations well (38.5%) (Fig. 3). In terms of the expectations for future measures, 42.8% of participants were more inclined to maintain zero-infection in society under the premise of ensuring normal life.

We also found that once infected with SARS-CoV-2, three most concerns of the participants were potential sequelae (67.3%), the negative impact on work or income due to isolation (57.6%), and troubles for others (57.4%), respectively. When the case fatality rate of SARS-CoV-2 was not higher than that of influenza, the control measures that the public were more willing to accept included wearing masks (86.0%) and free COVID-19 vaccination (73.2%), as shown in Fig. 3.

**Table 2:** Public’s cognition and overall attitude towards SARS-CoV-2 infection and control measures in China, August 2022

Possible Variables	Total Participants (*n* = 1391) (%)	Public Acceptance			Univariate logistic regression analysis	
		High (*n* = 572) (%)	Low (*n* = 819) (%)	*P*-Value ^1^	Odds Ratio (OR) and 95% Confidence Interval (95% CI)	*P*-Value ^2^
Fear of SARS-CoV-2 infection (scores), mean (standard deviations)	4.78 (3.19)	5.29 (3.33)	4.43 (3.04)	< 0.001 ***	-	-
Fear of SARS-CoV-2 infection category (scores)				< 0.001 ***		< 0.001 ***
0–4	573 (41.2)	200 (35.0)	373 (45.5)		1.748 (1.372, 2.228)	
5	293 (21.1)	118 (20.6)	175 (21.4)		1.390 (1.041, 1.857)	
6–10	525 (37.7)	254 (44.4)	271 (33.1)		Reference	
Perceived case fatality rate of SARS-CoV-2				< 0.001 ***		< 0.001 ***
Very low	376 (27.0)	103 (18.0)	273 (33.3)		12.490 (5.717, 27.287)	
Low	452 (32.5)	182 (31.8)	270 (33.0)		6.958 (3.201, 15.123)	
Average	411 (29.5)	193 (33.7)	218 (26.6)		5.468 (2.512, 11.902)	
High	124 (8.9)	71 (12.4)	53 (6.5)		3.610 (1.607, 8.110)	
Very high	28 (2.0)	23 (4.0)	5 (0.6)		Reference	
When the case fatality rate of SARS-CoV-2 drops to this level of influenza, I think the restrictions can be appropriately relaxed				< 0.001 ***		< 0.001 ***
2–3 times	167 (12.0)	55 (9.6)	112 (13.7)		3.557 (2.632, 4.807)	
The same	921 (66.2)	334 (58.4)	587 (71.7)		2.663 (2.186, 3.245)	
One-tenth	303 (21.8)	183 (32.0)	120 (14.7)		Reference	
COVID-19 vaccination status				0.046 *		0.009 **
No	39 (2.8)	10 (1.7)	29 (3.5)		Reference	
Yes	1352 (97.2)	562 (98.3)	790 (96.5)		0.489 (0.285, 0.839)	
Overall satisfaction of COVID-19 control measures				< 0.001 ***		< 0.001 ***
Dissatisfied	100 (7.2)	4 (0.7)	96 (11.7)		33.070 (16.126, 67.816)	
Average	244 (17.5)	42 (7.3)	202 (24.7)		7.773 (5.869, 10.295)	
Satisfied	464 (33.4)	178 (31.1)	286 (34.9)		2.282 (1.900, 2.740)	
Very satisfied	583 (41.9)	348 (60.8)	235 (28.7)		Reference	
Public’s expectations of future control measures				< 0.001 ***		< 0.001 ***
Continue strict control measures and continue to maintain zero-infection in society	193 (13.9)	146 (25.5)	47 (5.7)		Reference	
Continue to maintain zero-infection in society, but hope to ensure normal life	596 (42.8)	300 (52.4)	296 (36.1)		2.821 (2.152, 3.697)	
Relax control measures gradually	452 (32.5)	113 (19.8)	339 (41.4)		8.596 (6.451, 11.455)	
Fully relaxation as soon as possible	147 (10.6)	12 (2.1)	135 (16.5)		34.098 (20.719, 56.115)	
Others	3 (0.2)	1 (0.2)	2 (0.2)		5.705 (1.027, 31.687)	

*Note*: *P-*values comparing different groups were from t-test, chi-squared test and univariate logistic regression analysis. ^1^ Significance difference: *P* < 0.05. ^2^ Significance difference of univariate logistic regression analysis: *P* < 0.1. * *P* < 0.05. ** *P* < 0.01. *** *P* < 0.001. The “-” indicated no data

### Impact of control measures on public’s life and work

We have set up a total of eleven items related to the impact on public’s life (Table 3). The results showed that there were significant differences in ten items between participants with high acceptance and low acceptance (*P* < 0.05), except for increased daily expenses. Meanwhile, greater impact of control measures on life contributed to lower public acceptance (OR = 1.946, 95% CI: 1.529 to 2.476).

**Table 3:** Impact of COVID-19 control measures on public’s life, August 2022

Items	Total Participants (*n* = 1391) (%)	Public Acceptance			Univariate logistic regression analysis	
		High (*n* = 572) (%)	Low (*n* = 819) (%)	*P*-Value ^1^	Odds Ratio (OR) and 95% Confidence Interval (95% CI)	*P*-Value ^2^
1. Failure to take medicine in time				< 0.001 ***	-	-
Yes	630 (45.3)	206 (36.0)	424 (51.8)			
No	761 (54.7)	366 (64.0)	395 (48.2)			
2. Delay going to hospital				< 0.001 ***	-	-
Yes	803 (57.7)	285 (49.8)	518 (63.2)			
No	588 (42.3)	287 (50.2)	301 (36.8)			
3. Failure to receive timely diagnosis and treatment leading to exacerbation of the condition				< 0.001 ***	-	-
Yes	426 (30.6)	144 (25.2)	282 (34.4)			
No	965 (69.4)	428 (74.8)	537 (65.6)			
4. Cause other diseases or injuries				< 0.001 ***	-	-
Yes	403 (29.0)	131 (22.9)	272 (33.2)			
No	988 (71.0)	441 (77.1)	547 (66.8)			
5. Cause mental stress (such as depression, anxiety or restlessness)				< 0.001 ***	-	-
Yes	661 (47.5)	220 (38.5)	441 (53.8)			
No	730 (52.5)	352 (61.5)	378 (46.2)			
6. Reduce offline shopping				0.001 **	-	-
Yes	1009 (72.5)	387 (67.7)	622 (75.9)			
No	382 (27.5)	185 (32.3)	197 (24.1)			
7. Reduce gatherings				0.034 *	-	-
Yes	1227 (88.2)	492 (86.0)	735 (89.7)			
No	164 (11.8)	80 (14.0)	84 (10.3)			
8. Reduce travelling				0.002 **	-	-
Yes	1263 (90.8)	503 (87.9)	760 (92.8)			
No	128 (9.2)	69 (12.1)	59 (7.2)			
9. Increased daily expenses				0.411	-	-
Yes	504 (36.2)	200 (35.0)	304 (37.1)			
No	887 (63.8)	372 (65.0)	515 (62.9)			
10. Stay abroad due to isolation measures				0.001 **	-	-
Yes	460 (33.1)	160 (28.0)	300 (36.6)			
No	931 (66.9)	412 (72.0)	519 (63.4)			
11. Other inconveniences in life				< 0.001 ***	-	-
Yes	589 (42.3)	197 (34.4)	392 (47.9)			
No	802 (57.7)	375 (65.6)	427 (52.1)			
12. Total score group of impact on public’s life				< 0.001 ***		< 0.001 ***
3 and below	367 (26.4)	195 (34.1)	172 (21.0)		Reference	
4 and above	1024 (73.6)	377 (65.9)	647 (79.0)		1.946 (1.529, 2.476)	

*Note*: *P-*values comparing different groups were from chi-squared test and univariate logistic regression analysis

^1^ Significance difference: *P* < 0.05. ^2^ Significance difference of univariate logistic regression analysis: *P* < 0.1

* *P* < 0.05. ** *P* < 0.01. *** *P* < 0.001. The “-” indicated no data

There were five questions regarding the impact of control measures on public’s work in our questionnaire (see Table S1 in Additional file 1). Finally, the results showed that higher total score of the impact on work brought about lower acceptance among the employed participants (OR = 1.493, 95% CI: 1.155 to 1.931).

### Factors related to public’s acceptance

All the above factors that might affect public acceptance were analyzed separately via the univariate logistic model (Tables 1, 2 and 3), of which the statistically significant factors (*P* < 0.1) were further incorporated into the multiple logistic model.

The final results of the multiple logistic regression analysis showed that the young citizens aged < 18 (OR = 8.251, 95% CI: 2.009 to 33.889) and 18–29 (OR = 2.349 95% CI: 1.564 to 3.529), and participants whose household per capita monthly income were lower than 5000 yuan (OR = 1.512, 95% CI: 1.085 to 2.105) were more resistant to accepting control measures.

Moreover, the individuals who perceived that the CFR of SARS-CoV-2 was extremely low (OR = 6.010, 95% CI: 2.475 to 14.595) and that the restrictions could be appropriately relaxed when the CFR dropped to 2–3 times of the influenza (OR = 2.792, 95% CI: 1.939 to 4.023) were less willing to accept control measures. Similarly, participants who were dissatisfied with control measures (OR = 9.639, 95% CI: 4.425 to 20.998) and preferred to fully relax controls as soon as possible (OR = 13.571, 95% CI: 7.751 to 23.758) had even lower acceptability (Table 4).

By contrast, rural residents had a higher acceptance than urban residents (OR = 0.683, 95% CI: 0.473 to 0.987). Students (OR = 0.510, 95% CI: 0.276 to 0.941) and employees working in the public (OR = 0.417, 95% CI: 0.240 to 0.727) and private (OR = 0.562, 95% CI: 0.320 to 0.986) sectors showed better compliance than those who were unemployed or retired. Compared with the unvaccinated respondents, those who had received COVID-19 vaccination before were more obedient to the control measures (OR = 0.429, 95% CI: 0.228 to 0.809).

The model passed the Hosmer and Lemeshow test (*P* = 0.132), indicating that this model fit well. Collinearity test proved that there was no collinearity among all variables in the model (tolerance > 0.1, VIF < 5).

**Table 4:** Multiple logistic regression analysis of associated factors for public acceptance of control measures, August 2022

Possible Variables ^1^	Odds Ratio (OR) and 95% Confidence Interval (95% CI)	*P*-Value
Age (years)		< 0.001 ***
< 18	8.251 (2.009, 33.889)	
18–29	2.349 (1.564, 3.529)	
30–49	1.319 (0.928, 1.874)	
≥ 50	Reference	
Residency		0.042 *
Rural	0.683 (0.473, 0.987)	
Urban	Reference	
Occupation		0.012 *
Public sector	0.417 (0.240, 0.727)	
Private sector	0.562 (0.320, 0.986)	
Self-employed or businessman	0.608 (0.324, 1.140)	
Students	0.510 (0.276, 0.941)	
Unemployed or retired	Reference	
Household per capita monthly income (yuan)		0.001 **
< 5000	1.512 (1.085, 2.105)	
5000–11,999	0.949 (0.713, 1.263)	
12,000–20,000	1.312 (0.953, 1.807)	
> 20,000	Reference	
Perceived case fatality rate of SARS-CoV-2		< 0.001 ***
Very low	6.010 (2.475, 14.595)	
Low	4.301 (1.800, 10.276)	
Average	3.994 (1.683, 9.476)	
High	3.121 (1.271, 7.6620	
Very high	Reference	
When the case fatality rate of SARS-CoV-2 drops to this level of influenza, I think the restrictions can be appropriately relaxed		< 0.001 ***
2–3 times	2.792 (1.939, 4.023)	
The same	1.827 (1.448, 2.306)	
One-tenth	Reference	
COVID-19 vaccination status		0.009 **
No	Reference	
Yes	0.429 (0.228, 0.809)	
Overall satisfaction of COVID-19 control measures		< 0.001 ***
Dissatisfied	9.639 (4.425, 20.998)	
Average	4.082 (2.993, 5.567)	
Satisfied	1.653 (1.340, 2.039)	
Very satisfied	Reference	
Public’s expectations of future control measures		< 0.001 ***
Continue strict control measures and continue to maintain zero-infection in society	Reference	
Continue to maintain zero-infection in society, but hope to ensure normal life	2.661 (1.974, 3.586)	
Relax control measures gradually	6.140 (4.383, 8.599)	
Fully relaxation as soon as possible	13.571 (7.751, 23.758)	
Others	3.637 (0.561, 23.556)	

*Note*: ^1^ Only variables that were statistically significant (*P* < 0.05) in multiple logistic regression analysis were listed in this table, and variables with *P* > 0.05 (such as educational qualification) were shown in Additional File 1 for details

* *P* < 0.05. ** *P* < 0.01. *** *P* < 0.001

## Discussion

COVID-19 in China was aggressive at the beginning and gradually stabilized after it was effectively controlled. Faced with the changing trend of COVID-19 currently and the emergence of various mutants, such as Omicron variant, the prevention and control strategies have also changed accordingly. As of August 2022, China’s preventive strategy of COVID-19 was no longer just an emergency control measure at the beginning, but a “dynamic COVID-zero strategy” in the normalization stage. In the context of Omicron, only by integrating the demands of the public into scientific control measures, can the government better ensure the implementation of measures and control the epidemic more effectively [21].

Understanding the public’s acceptance of control measures was thus crucial for informing control strategies of COVID-19. Our results showed that 58.9% of the study participants were less acceptive of the control measures while 41.1% were highly acceptive, which was completely inconsistent with the conclusion of a previous research [22]. With the emergence of SARS-CoV-2 variants, especially the Omicron variant, the public had also gained a deeper understanding of COVID-19 development, which might account for the change in public attitudes towards control measures.

In this survey, young people showed lower acceptance of control measures, which was similar to a study in Saudi Arabia [23]. Meanwhile, a French study also found that the pressure of study or work, and the pursuit of personal freedom and rich entertainment life made it more difficult for the young to accept strict control measures [24]. Unexpectedly, the rural residents (vs. urban residents) and undereducated population (vs. master’s degree or above), especially the high-school qualification or below, were more acceptive during the Omicron-dominated period. Relatively speaking, their daily lives were unaffected and unconstrained, but they were generally deficient in comprehensive and correct knowledge of COVID-19 [25–29]. For this reason, they might be more afraid of contracting SARS-CoV-2 and the resulting medical costs, and consequently preferred to accept control measures to avoid infection.

After the adjustment of multivariate analysis, the potential factor, occupation, had both statistical and practical significance. Despite the emotional toll [30] or elevated workload [31, 32] from all walks of life during the pandemic, when compared with the financial stress and anxiety resulted from employment disruptions (the jobless or retired), students and those who were still employed had less financial burden and were better trained, so they showed more willingness to cooperate with control measures [29, 33, 34]. Likewise, public acceptance would be low when the household per capita monthly income fell below 5000 yuan. Since COVID-19 crisis, the elevated-risk of unemployment and increased daily expenses resulted in huge life pressure [35]. Unsurprisingly, participants who previously had a history of lockdown or controlled areas showed greater opposition to the control measures. Long-term movement restrictions and distance rules led to restrictions on personal freedom and more inconveniences in public’s normal life and work [36].

Similar to the findings of Constant et al. [24], in our study, different cognitions and overall attitudes towards COVID-19 control measures were also related to public acceptance. Those who perceived less fear of SARS-CoV-2 infection and believed that the CFR was extremely low were less acceptive. Moreover, study participants who reputed that restrictions could be relaxed when the CFR dropped to 2–3 times of influenza expressed lower compliance, which might be related to the traits of high infectivity but low lethality of the Omicron variant [37, 38].

Our results also indicated that participants who were dissatisfied with the control measures or expected fully relaxation as soon as possible were much less supportive. It has been reported that quarantine measures have caused inconveniences in public’s daily life, and even a negative psychological state to the public [39, 40], which was also responsible for the decline in public acceptance. Conversely, respondents who had received COVID-19 vaccination before showed a more acceptive attitude towards control measures, which represented that the public actively responded to the call and were willing to build an immune barrier via vaccination.

Restricted by objective conditions, our study inevitably had limitations. Due to the lockdown requirements, only web-based survey could be conducted during the study period. Moreover, since the questionnaire was originally released in Guangdong Province, most of the respondents were concentrated in Guangdong. Considering China’s vast territory, the strictness of policy enforcements were quite different in Chinese local governments, which might also influence the citizens’ acceptance.

Nonetheless, the public who participated in this survey were spread across China, with all densely populated regions (such as Guangdong Province in the south, Henan Province in the north, Beijing, the capital of China, and the Xinjiang Uyghur Autonomous Region in the west) involved and only few sparsely populated regions uninvolved. The impact of enforcement intensity in different regions on public acceptance was also revealed due to the breadth of the geographical coverage in our study. For instance, Guangdong was the typical province in China where local governments had effectively implemented control measures and withstood the wave after wave of COVID-19 outbreaks. Meanwhile, China’s first pioneer city to fully ease controls in 2022 was also located in Guangdong, whose actual situation could definitely provide a crucial reference for comprehensively assessing the public acceptance of control measures in various regions of China.

In fact, our findings could mutually corroborate China’s subsequent decision to further optimize and fully loosen restrictions in December 2022 [17], which well reflected the accuracy and foresightedness of this study previously investigated in August 2022. Most importantly, this study could have well included questions regarding the past experiences of COVID-19 pandemic in China. The past experiences played a significant role in deciding present mental and behavioral state of the respondents. Without it, we cannot make cross-sectional comment specifically on Omicron-dominant era in China. Hence, this study has important practical significance and great empirical value in a specific era, which can provide more references for formulating humane public health strategies in the future and improving public acceptance to control the epidemic more scientifically and effectively.

## Conclusion

In this cross-sectional study, more than half (58.9%) of the Chinese public had a low acceptance of COVID-19 control measures during the Omicron-dominant era, which varied in accordance with their different demographic characteristics, cognition and overall attitudes. In general, the control measures that struck a balance between public safety and individual freedom would be more acceptable and thus more sustainable while controlling the spread of the pandemic.

### Electronic supplementary material

Below is the link to the electronic supplementary material.

Supplementary Material 1

## Data Availability

The datasets used and/or analyzed during the current study are available from the corresponding author on reasonable request.

## Abbreviations

COVID-19: coronavirus disease 2019
CFR: case fatality rate
SARS-CoV-2: severe acute respiratory syndrome coronavirus-2
SARS: severe acute respiratory syndrome
QR code: quick response code
qPCR: quantitative real-time polymerase chain reaction
SD: standard deviation
IQR: interquartile range
OR: odds ratio
CI: 95% confidence interval
