# Supplementary material for: Public acceptance of COVID-19 control measures and associated factors during Omicron-dominant period in China: a cross-sectional survey
Source: BMC Public Health. 2024 Feb 21;24:543. doi: 10.1186/s12889-024-17646-3 (PMC10882874; doi:10.1186/s12889-024-17646-3)
Supplement: Supplementary file 1 — Supplementary Material 1 [file 12889_2024_17646_MOESM1_ESM.docx]

**Supplemental Materials**

We set up five items about the impact of control measures on public’s work in the questionnaire. The results suggested that between participants with high and low acceptance, the public acceptance was significantly associated with decreased salary, unsatisfactory work progress and the total score of the impact on work (*P*<0.05). Moreover, the results showed that higher total score of the impact on work brought about lower acceptance among the employed participants (OR=1.493, 95% CI: 1.155 to 1.931).

Table S1 Impact of COVID-19 control measures on public’s work, August 2022

| **Items** | **Total**  **Participants**  **(n=1023) (%)** | **Public Acceptance** | | | **Univariate logistic regression analysis** | |
| --- | --- | --- | --- | --- | --- | --- |
|  |  | **High**  **(n=429) (%)** | **Low**  **(n=594) (%)** | ***P*-Value ^1^** | **Odds Ratio (OR) and**  **95% Confidence Interval (95% CI)** | ***P*-Value ^2^** |
| 1. Increased workload |  |  |  | 0.279 | - | - |
| Yes | 609 (59.5) | 247 (57.6) | 362 (60.9) |  |  |  |
| No | 414 (40.5) | 182 (42.4) | 232 (39.1) |  |  |  |
| 1. Unemployment |  |  |  | 0.413 | - | - |
| Yes | 200 (19.6) | 89 (20.7) | 111 (18.7) |  |  |  |
| No | 823 (80.4) | 340 (79.3) | 483 (81.3) |  |  |  |
| 1. Increased frequency of occupational replacement |  |  |  | 0.996 | - | - |
| Yes | 174 (17.0) | 73 (17.0) | 101 (17.0) |  |  |  |
| No | 849 (83.0) | 356 (83.0) | 493 (83.0) |  |  |  |
| 1. Decreased salary |  |  |  | 0.009 ** | - | - |
| Yes | 597 (58.4) | 230 (53.6) | 367 (61.8) |  |  |  |
| No | 426 (41.6) | 199 (46.4) | 227 (38.2) |  |  |  |
| 1. Unsatisfactory work progress |  |  |  | <0.001 *** | - | - |
| Yes | 612 (59.8) | 222 (51.7) | 390 (65.7) |  |  |  |
| No | 411 (40.2) | 207 (48.3) | 204 (34.3) |  |  |  |
| 1. Total score group of impact on public’s work |  |  |  | 0.002 ** |  | 0.002 ** |
| 1 and below | 376 (36.8) | 181 (42.2) | 195 (32.8) |  | Reference |  |
| 2 and above | 647 (63.2) | 248 (57.8) | 399 (67.2) |  | 1.493 (1.155, 1.931) |  |

Note: *P-*values comparing different groups were from chi-squared test and univariate logistic regression analysis.

^1^ Significance difference: *P*<0.05. ^2^ Significance difference of univariate logistic regression analysis: *P*<0.1.

** *P*<0.01. *** *P*<0.001. The “-” indicated no data.

After multiple logistic regression analysis, the factors that were not statistically significant (*P*>0.05) were listed below for details.

Table S2 Multiple logistic regression analysis of associated factors for public acceptance of control measures, August 2022

| **Possible Variables ^1^** | **Odds Ratio (OR) and**  **95% Confidence Interval (95% CI)** | ***P*-Value** |
| --- | --- | --- |
| Educational qualification |  | 0.151 |
| High school or below | 0.675 (0.453, 1.005) |  |
| Universities and colleges | 0.893 (0.720, 1.106) |  |
| Master degree or above | Reference |  |
| History of a lockdown area |  | 0.056 |
| No | Reference |  |
| Yes | 1.388 (0.992, 1.943) |  |
| History of a controlled area |  |  |
| No | Reference | 0.435 |
| Yes | 0.907 (0.709, 1.159) |  |
| Fear of SARS-CoV-2 infection category (scores) |  | 0.378 |
| 0-4 | 0.850 (0.676, 1.069) |  |
| 5 | 0.917 (0.715, 1.177) |  |
| 6-10 | Reference |  |
| Total score group of impact on public’s life |  | 0.405 |
| 3 and below | Reference |  |
| 4 and above | 1.093 (0.887, 1.347) |  |

Note: ^1^ Variables with *P*>0.05 in the multiple logistic regression analysis.
